# Prenatal parental tobacco smoking, gene specific DNA methylation, and newborns size: the Generation R study

**DOI:** 10.1186/s13148-015-0115-z

**Published:** 2015-08-11

**Authors:** Marieke I. Bouwland-Both, Nina H. van Mil, Catharina P. Tolhoek, Lisette Stolk, Paul H. C. Eilers, Michael M. P. J. Verbiest, Bastiaan T. Heijmans, André G. Uitterlinden, Albert Hofman, Marinus H. van Ijzendoorn, Liesbeth Duijts, Johan C. de Jongste, Henning Tiemeier, Eric A. P. Steegers, Vincent W. V. Jaddoe, Régine P. M. Steegers-Theunissen

**Affiliations:** The Generation R Study Group, Erasmus MC, University Medical Centre Rotterdam, Rotterdam, The Netherlands; Department of Obstetrics and Gynecology, Erasmus MC, University Medical Centre, Ee-building Room 2271a, PO Box 1738, 3000 DR Rotterdam, The Netherlands; Department of Child and Adolescent Psychiatry/Psychology, Erasmus MC, University Medical Centre, Rotterdam, The Netherlands; Department of Internal Medicine, Erasmus MC, University Medical Centre, Rotterdam, The Netherlands; Department of Biostatistics, Erasmus MC, University Medical Centre, Rotterdam, The Netherlands; Department of Molecular Epidemiology, Leiden University Medical Centre, Leiden, The Netherlands; Department of Epidemiology, Erasmus MC, University Medical Centre, Rotterdam, The Netherlands; School for Pedagogical and Educational Sciences, Erasmus MC, University Medical Centre, Rotterdam, The Netherlands; Department of Pediatrics, Erasmus Medical Center, Rotterdam, The Netherlands

**Keywords:** Cigarettes, Cord blood, DNA methylation, Epigenetic epidemiology, *H19*, *IGF2DMR*, Maternal tobacco smoking, Paternal tobacco smoking

## Abstract

**Background:**

Deleterious effects of prenatal tobacco smoking on fetal growth and newborn weight are well-established. One of the proposed mechanisms underlying this relationship is alterations in epigenetic programming. We selected 506 newborns from a population-based prospective birth cohort in the Netherlands. Prenatal parental tobacco smoking was assessed using self-reporting questionnaires. Information on birth outcomes was obtained from medical records. The deoxyribonucleic acid (DNA) methylation of the growth genes *IGF2DMR* and *H19* was measured in newborn umbilical cord white blood cells. Associations were assessed between parental tobacco smoking and DNA methylation using linear mixed models and adjusted for potential confounders.

**Results:**

The DNA methylation levels of IGF2DMR and H19 in the non-smoking group were median (90 % range), 54.0 % (44.6–62.0), and 30.0 % (25.5–34.0), in the first trimester only smoking group 52.2 % (44.5–61.1) and 30.8 % (27.1–34.1), and in the continued smoking group 51.6 % (43.9–61.3) and 30.2 % (23.7–34.8), respectively. Continued prenatal maternal smoking was inversely associated with *IGF2DMR* methylation (*β* = −1.03, 95 % CI −1.76; −0.30) in a dose-dependent manner (*P*-trend = 0.030). This association seemed to be slightly more profound among newborn girls (*β* = −1.38, 95 % CI −2.63; −0.14) than boys (*β* = −0.72, 95 % CI −1.68; 0.24). *H19* methylation was also inversely associated continued smoking <5 cigarettes/day (*β* = −0.96, 95 % CI −1.78; −0.14). Moreover, the association between maternal smoking and newborns small for gestational age seems to be partially explained by *IGF2DMR* methylation (*β* = −0.095, 95 % CI −0.249; −0.018). Among non-smoking mothers, paternal tobacco smoking was not associated with *IGF2DMR* or *H19* methylation.

**Conclusions:**

Maternal smoking is inversely associated with *IGF2DMR* methylation in newborns, which can be one of the underlying mechanisms through which smoking affects fetal growth.

**Electronic supplementary material:**

The online version of this article (doi:10.1186/s13148-015-0115-z) contains supplementary material, which is available to authorized users.

## Background

Impaired fetal growth and low birth weight increase the risk of short and long-term morbidity and mortality [1, 2]. A suboptimal prenatal environment contributes to fetal endocrine and metabolic adaptations with permanent effects [3, 2]. An important modifiable and adverse prenatal exposure is tobacco smoking by parents-to-be. The deleterious effects of maternal tobacco smoking on fetal growth and newborn weight are well-established [4]. The underlying mechanisms are not fully known but include vasoconstriction, reduced placental and fetal perfusion, and hypoxia [5]. Accumulating data suggest that derangements in epigenetic fetal and placental (re)programming may be one of the underlying mechanisms explaining the association between prenatal tobacco smoking and impaired fetal growth [6].

Epigenetic alterations are changes in gene expression potential that are not directly related to the deoxyribonucleic acid (DNA) sequence itself. DNA methylation modifications are one of the best understood epigenetic mechanisms [7]. During pregnancy, cells undergo major epigenetic reprogramming making them more susceptible for derangements during this period [7]. Both animal and human studies have shown that changes in the prenatal environment can lead to variations in epigenetic profiles [8–10]. Previous studies have shown global and gene specific differences in DNA methylation in different tissues in children prenatally exposed to tobacco smoking [11–13]. Insulin growth factor 2 (*IGF2*) and *H19* are maternally and paternally imprinted genes located next to each other, playing an important role during fetal growth and development (Additional file 1: Figure S1) [14–17]. Moreover, the *IGF2*/*H19* locus is a well-studied region that is known to be regulated through epigenetic mechanisms and sensitive to environmental exposures [9]. Previous research demonstrated that men may be more susceptible to detrimental early prenatal exposures than women [18].

Less is known about the influence of prenatal paternal tobacco smoking on gene-specific DNA methylation in newborns. Moreover, information on paternal tobacco smoking can be used to elucidate whether an effect on DNA methylation in newborns is due to the direct intrauterine effect of maternal smoking.

From this background, we aim to study whether prenatal parental tobacco smoking is associated with DNA methylation of *IGF2* differentially methylated region (*DMR*) and *H19* in umbilical white cord blood cells of newborns, with a focus on differences in sensitivity between genders. Furthermore, we examined mediation of DNA methylation in the association between tobacco smoking and the risk of being born small for gestational age (SGA).

## Results

Maternal and newborns characteristics are presented in Table 1. Of the included mothers, 9.3 % (*n* = 47) reported tobacco smoking only in the first trimester and 24.7 % (*n* = 125) continued smoking during pregnancy. Mothers who continued tobacco smoking were younger, lower educated, and less often used a folic acid supplement during the periconception period than mothers who never smoked during pregnancy. Paternal tobacco smoking was more frequent in families with mothers who continued smoking (72.8 % in continued smoking versus 37.4 % in non-smoking mothers). Median (90 % range) birth weights of newborns from non-smokers, first trimester only and continued smoking mothers were 3500 (2536–4383), 3410 (2154–4219), and 3195 (2348–4081) grams. The median (90 % range) DNA methylation levels of *IGF2DMR* and *H19* were 53.2 % (44.3–61.3) and 30.1 % (25.6–34.1), respectively. *IGF2DMR* and *H19* were correlated (Pearsons *r* = 0.147, *P* value = 0.001). The DNA methylation levels of *IGF2DMR* and *H19* in the non-smoking group were median (90 % range), 54.0 % (44.6–62.0), and 30.0 % (25.5–34.0), in the first trimester only smoking group 52.2 % (44.5–61.1) and 30.8 % (27.1–34.1), and in the continued smoking group 51.6 % (43.9–61.3) and 30.2 % (23.7–34.8), respectively. In addition, the methylation levels of *IGF2DMR* and *H19* were plotted according to each of the smoking categories (Additional file 2: Figure S2).

**Table 1:** Baseline characteristics

Characteristic	Prenatal maternal tobacco smoking					
	No (*n* = 334)	First trimester only (*n* = 47)	Continued			
			All (*n* = 125)	<5 cigarettes per day (*n* = 41)	≥5 cigarettes per day (*n* = 56)	P value^c^
*Maternal*						
Age at intake (years)^a, b^	31.5 (22.5–38.1)	30.5 (20.1–38.3)	28.5 (19.0–37.7)	29.4 (20.0–37.5)	28.4 (18.4–39.9)	<0.001
Body mass index at intake (kg/m^2^)^a, b^	23.1 (19.5–32.2)	23.2 (19.6–33.1)	23.7 (18.2–32.5)	22.8 (18.2–33.6)	24.8 (18.0–33.9)	NS
Education, *n* (%)						<0.001
Primary education	6 (1.8)	4 (8.5)	12 (9.6)	0 (0)	10 (17.9)	
Secondary education	134 (40.1)	21 (44.7)	78 (62.4)	23 (56.1)	34 (60.7)	
Higher education	193 (57.8)	22 (46.8)	33 (26.4)	17 (41.5)	11 (19.6)	
Missing	1 (0.3)	0 (0.0)	2 (1.6)	1 (2.4)	1 (1.8)	
Parity (%)						NS
0	220 (65.9)	34 (72.3)	82 (65.6)	28 (68.3)	35 (62.5)	
≥1	114 (34.1)	13 (27.7)	43 (34.4)	13 (31.7)	21 (37.5)	
Folic acid supplement use during pregnancy, *n* (%)						<0.001
Start preconception	188 (56.3)	18 (38.3)	29 (23.2)	11 (26.8)	15 (26.8)	
Start postconception	84 (25.1)	19 (40.4)	36 (28.8)	13 (31.7)	13 (23.2)	
No	25 (7.5)	7 (14.9)	33 (26.4)	10 (24.4)	14 (25)	
Missing	37 (11.1)	3 (6.4)	27 (21.6)	7 (17.1)	14 (25)	
Paternal smoking, *n* (%)	125 (37.4)	28 (59.6)	91 (72.8)	14 (34.1)	9 (16.1)	<0.001
	<5 cigarettes per day	57 (17.1)	9 (19.1)	17 (13.6)	7 (17.1)	17 (30.4)
	≥5 cigarettes per day	67 (20.1)	18 (38.3)	73 (58.4)	4 (9.8)	39 (69.6)
*Newborns*						
Boys, *n* (%)	195 (58.4)	21 (44.7)	81 (64.8)	26 (63.4)	34 (60.7)	NS
Birth weight^a, b^	3500 (2536–4383)	3410 (2154–4219)	3195 (2348–4081)	3175 (2663–4252)	3210 (2334–4150)	<0.001
Gestational age at birth^a, b^	40.3 (37.6–42.0)	40.3 (36.5–41.9)	40.1 (37.1–42.5)	40.6 (37.0–42.9)	40.2 (36.9–42.3)	NS
*IGF2DMR* methylation (%)^a^	54.0 (44.6–62.0)	52.2 (44.5–61.1)	51.6 (43.9–61.3)	52.0 (44.0–61.2)	51.1 (41.2–63.9)	0.033
*H19* methylation (%)^1^	30.0 (25.5–34.0)	30.8 (27.1–34.1)	30.2 (23.7–34.8)	29.8 (21.5–33.0)	30.2 (23.1–37.0)	NS

^a^Values are presented as median (90 % range) or as number (%)

^b^Missings; age at intake (*n* = 0), body mass index at intake (*n* = 1), gender (*n* = 0) birth weight (*n* = 0), gestational age at birth (*n* = 0)

^c^ANOVA and chi-square tests are used to test differences between the different smoking categories

The associations between parental tobacco smoking and DNA methylation of *IGF2DMR* and *H19* are shown in Table 2. Although *IGF2DMR* methylation of mothers who smoked only during the first trimester showed a tendency to be lower compared to non-smokers, no significant differences were observed. However, continued prenatal smoking was associated with lower *IGF2DMR* methylation (crude model, *β* = −1.14, 95 % CI −1.81; −0.47, *P* value = 0.001; adjusted model, *β* = −1.03, 95 % CI −1.76; −0.30, *P* value = 0.006). Expressed as relative to the standard deviation, the difference found in the adjusted model corresponds with a standardized effect size in DNA methylation of −0.13 standard deviation score (SDS). Of the mothers who continued smoking, 77.6 % (*n* = 97) provided information concerning the amount of cigarettes they smoked, of which 42.3 % (*n* = 41) reported to have smoked less than five cigarettes per day and 57.7 % (*n* = 56) reported to have smoked five or more cigarettes per day. No significant association was found between the amount of cigarettes and *IGF2DMR* methylation. However, a dose-dependent association of the number of cigarettes smoked was assessed in mothers who continued smoking (*P* value for trend = 0.030). Although no association was observed between mothers who continued smoking and *H19* methylation, a significantly inverse association was revealed in mothers smoking up to five cigarettes per day (crude model, −0.90, 95 % CI −1.70; −0.11, *P* value = 0.026; adjusted model, *β* = −0.96, 95 % CI −1.78; −0.14, *P* value = 0.021). Expressed relative to the standard deviation, the difference found in the adjusted model corresponds with a standardized effect size in DNA methylation of −0.13 SDS. No significant associations were observed in mothers who reported to have smoked five or more cigarettes per day with *H19* methylation. Among mothers who did not smoke during pregnancy, information on prenatal paternal tobacco smoking was provided by 99.4 % of the mothers (*n* = 332/334). Of the fathers who smoked, 45.6 % (*n* = 57/125) smoked less than five cigarettes per day and 53.6 % (*n* = 67/125) smoked five or more cigarettes per day. No association was observed between paternal smoking and *IGF2DMR* or *H19* methylation. We repeated the analyses after exclusion of the attention deficit hyperactivity disorder (ADHD) cases (see Additional file 3: Table S3), which did not change substantially the effect estimates.

**Table 2:** Prenatal parental tobacco smoking habits and DNA methylation

		*IGF2DMR* methylation			*H19* methylation		
		bèta^a^	95 % CI	*P* value	bèta^a^	95 % CI	*P* value
*Model 1: adjusted for correlations between CpG sites, bisulphite batch, gestational age at birth*							
Maternal tobacco smoking							
No (*n* = 334)		Reference			Reference		
First trimester only (*n* = 47)		−0.68	−1.67; 0.31	0.178	0.61	−0.13; 1.35	0.108
Continued smoking, all (*n* = 125)		−1.14	−1.81; −0.47	0.001	−0.24	−0.75; 0.26	0.348
	<5 cigarettes per day (*n* = 41)	−1.06	−2.13; −0.00	0.050	−0.90	−1.70; −0.11	0.026
	≥5 cigarettes per day (*n* = 56)	−1.12	−2.06; −0.18	0.019	−0.13	−0.82; 0.56	0.703
* P* for trend		0.006			0.298		
Paternal tobacco smoking							
No (*n* = 207)		Reference			Reference		
Yes (*n* = 125)		−0.24	−0.96; 0.48	0.519	−0.10	−0.61; 0.41	0.707
	<5 cigarettes per day (*n* = 57)	−0.26	−1.22; 0.70	0.597	−0.19	−0.87; 0.50	0.592
	≥5 cigarettes per day (*n* = 67)	−0.16	−1.06; 0.73	0.719	−0.04	−0.68; 0.60	0.893
* P* for trend		0.643			0.795		
*Model 2: model 1 + maternal characteristics (age, educational level, parity, BMI, periconception folic acid supplement use) and fetal gender*							
Maternal tobacco smoking							
No (*n* = 334)		Reference			Reference		
First trimester only (*n* = 47)		−0.70	−1.71; 0.31	0.175	0.56	−0.20; 1.32	0.146
Continued smoking, all (*n* = 125)		−1.03	−1.76; −0.30	0.006	−0.37	−0.94; 0.20	0.195
	<5 cigarettes per day (*n* = 41)	−1.01	−1.89; 0.10	0.069	−0.96	−1.78; −0.14	0.021
	≥5 cigarettes per day (*n* = 56)	−0.89	−2.10; 0.08	0.079	−0.20	−0.93; 0.54	0.602
* P* for trend		0.030			0.242		
Paternal tobacco smoking							
No (*n* = 207)		Reference			Reference		
Yes (*n* = 125)		−0.17	−0.91; 0.58	0.663	−0.23	−0.75; 0.30	0.393
	<5 cigarettes per day (*n* = 57)	−0.20	−1.18; 0.77	0.681	−0.23	−0.92; 0.45	0.502
	≥5 cigarettes per day (*n* = 67)	−0.07	−1.01; 0.88	0.887	−0.25	−0.91; 0.42	0.463
* P* for trend		0.809			0.395		

Results from linear mixed model analyses with maternal and paternal prenatal tobacco smoking as independent variable and DNA methylation as dependent variable

^a^Analyses were performed with square root transformed methylation data and values are presented as regression coefficients (95 % confidence interval). Analyses on paternal smoking were restricted to non-smoking mothers

Table 3 shows the associations between maternal tobacco smoking and DNA methylation, stratified by gender. In mothers who smoked during the first trimester, an inverse association among newborn girls was shown with *IGF2DMR* methylation (crude model, *β* = −1.40, 95 % CI −2.79; −0.01, *P* value = 0.048), which attenuated in the adjusted model (*β* = −1.25, 95 % CI −2.71; 0.21, *P* value = 0.093). This association was not observed among boys. In addition, first trimester smoking was positively associated with *H19* methylation among girls but lost significance after adjustment (crude model, *β* = 1.04, 95 % CI 0.04; 2.03, *P* value = 0.041; adjusted model, *β* = 1.00, 95 % CI −0.05; 2.03, *P* value = 0.063). The association between continued maternal tobacco smoking and *IGF2DMR* methylation was slightly more profound among girls (crude model, *β* = −1.46, 95 % CI −2.59; −0.33, *P* value = 0.011; adjusted model, *β* = −1.38, 95 % CI −2.63; −0.14, *P* value = 0.029) than in boys (crude model, *β* = −0.89, 95 % CI −1.73; −0.06, *P* value = 0.037; adjusted model, *β* = −0.72, 95 % CI −1.68; 0.24, *P* value = 0.142).

**Table 3:** Prenatal maternal tobacco smoking and DNA methylation, stratified by gender

	*IGF2DMR* methylation			*H19* methylation		
	bèta^a^	95 % CI	*P* value	bèta^a^	95 % CI	*P* value
*Model 1: adjusted for correlations between CpG sites, bisulphite batch, gestational age at birth*						
Boys						
No (*n* = 195)	Reference			Reference		
First trimester only (*n* = 21)	0.12	−1.33; 1.57	0.870	0.18	−0.95; 1.31	0.754
Continued smoking, all (*n* = 81)	−0.89	−1.73; −0.06	0.037	−0.41	−1.07; 0.25	0.222
* P* for trend	0.044			0.249		
Girls						
No (*n* = 139)	Reference			Reference		
First trimester only (*n* = 26)	−1.40	−2.79; −0.01	0.048	1.04	0.04; 2.03	0.041
Continued smoking, all (*n* = 44)	−1.46	−2.59; −0.33	0.011	−0.10	−0.90; 0.71	0.816
* P* for trend	0.005			0.817		
*Model 2: model 1 + maternal characteristics (age, educational level, parity, BMI, periconception folic acid supplement use)*						
Boys						
No (*n* = 195)	Reference			Reference		
First trimester only (*n* = 21)	0.15	−1.32; 1.61	0.845	0.14	−1.00; 1.28	0.810
Continued smoking, all (*n* = 81)	−0.72	−1.68; 0.24	0.142	−0.69	1.45; 0.08	0.078
* P* for trend	0.166			0.098		
Girls						
No (*n* = 139)	Reference			Reference		
First trimester only (*n* = 26)	−1.25	−2.71; 0.21	0.093	1.00	−0.05; 2.03	0.063
Continued smoking, all (*n* = 44)	−1.38	−2.63; −0.14	0.029	−0.05	−0.93; 0.83	0.910
* P* for trend	0.018			0.813		

Results from linear mixed model analyses with maternal and parental tobacco smoking as independent variable and DNA methylation as dependent variable, stratified by gender

^a^Analyses were performed with square root transformed methylation data and values are presented as regression coefficients (95 % confidence interval)

**Fig. 1:**
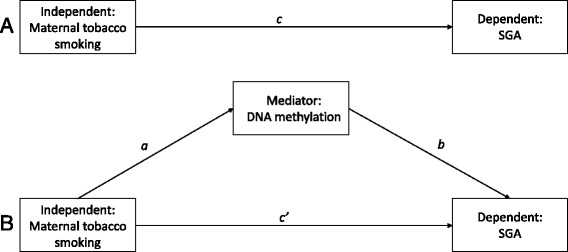
Graphical representation of proposed mediation effect. Panel **a** illustrates the total effect (path c). Panel **b** represents the mediation design. The direct effect is depicted as path c’. The indirect effect (ab) is defined as the product of path a and path b

**Fig. 2:**
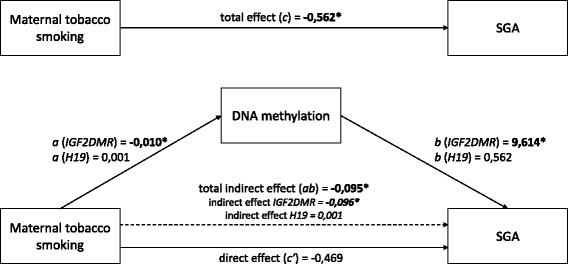
Mediation analyses. Values represent unstandardized beta’s. *P < 0.05

Associations between maternal tobacco smoking, the risk of a newborn being SGA, and gene-specific DNA methylation were analyzed using mediation analyses. We hypothesized that epigenetic alterations can mediate the association between maternal prenatal tobacco smoking and impaired fetal growth (Fig. 1). The indirect effect, defined as the product of the coefficient for the effect of maternal tobacco smoking on DNA methylation (path a), and the coefficient for the effect of DNA methylation on the risk of a newborns being SGA (path b), was calculated (Fig. 2). The observed significant effect (*β* = −0.095, 95 % CI −0.249; −0.018) substantiates the notion that the association between maternal prenatal smoking and the risk of a newborn being SGA is partially mediated through *IGF2DMR* methylation. The remaining direct effect of smoking on SGA (c’) seems to be explained by the slightly younger age and lower educational level of the smoking mother. No use of a folic acid supplement, higher body mass index (BMI) of the mother, nulliparity, and the male gender of the newborn did not significantly explain this effect.

## Discussion

### Main findings

In 506 newborns, derived from a population-based birth cohort, we examined whether parental tobacco smoking is associated with *IGF2DMR* and *H19* methylation in umbilical white cord blood cells. After adjustment for confounders, continued maternal prenatal tobacco smoking was associated in a dose-dependent manner with decreased *IGF2DMR* methylation in newborns. This association was stronger in newborn girls than boys. Decreased *H19* methylation was also observed in mothers who continued to smoke <5 cigarettes/day. Prenatal paternal smoking was not associated with *IGF2DMR* or *H19* methylation. Furthermore, the association between maternal smoking and the increased risk of being SGA may be partially mediated through *IGF2DMR* methylation.

### Interpretation

Prenatal maternal tobacco smoking has been recognized as an important and modifiable detrimental exposure and has repeatedly been associated with disease [4, 19, 5]. Several studies have investigated associations between maternal prenatal smoking and global and gene-specific DNA methylation [20, 6]. Epigenome-wide methylation analyses revealed methylation changes at birth in newborns whose mother had smoked during pregnancy [21, 22]. Others investigated DNA methylation differences at the *IGF2*/*H19* locus in relation to prenatal exposure to maternal tobacco smoking but found higher *IGF2DMR* methylation levels in boys born to smokers compared to non-smokers (+4.7 % *IGF2DMR* methylation) [15]. The region analyzed by Murphy et al. for the IGF2DMR included three CpG dinucleotides upstream of exon 3 (chr 11p15.5; CpG site 1, 2,169,518; CpG site 2, 2,169,515; and CpG site 3, 2,169,499; NCBI Human Genome Build 37/hg19) which overlapped with the region analyzed in our study (chr 11p15.5; CpG site 3, 2,169,709; CpG site 4, 2,169,660; CpG site 6/7, 2,169,518/2,169,515; NCBI Human Genome Build 37/hg19) [15]. The third CpG site analyzed by Murphy et al. overlapped with our eighth CpG site, which was excluded due to technical issues (see Additional file 4: Table S4) [15]. For the analyses of H19 DMR, the regions did not overlap. Other detrimental exposures, such as prenatal exposure to famine, have been previously linked to lower *IGF2DMR* methylation (−5.2 % *IGF2DMR* methylation when periconception exposure to famine), whereas advantageous exposures, such as periconceptional folic acid supplement use, have been associated with increased methylation (+4.5 % *IGF2DMR* methylation) [9, 8]. Our findings are also consistent with Guerrero-Preston et al., who also reported an inverse association between global DNA methylation and the nicotine biomarker cotinine however without stratification for gender [13]. In a previous study performed in the same population, we showed that SGA was associated with −1.13 % lower *IGF2DMR* methylation [17]. However, further research is warranted to further elucidate this difference.

In our study, the inverse association between maternal tobacco smoking from the first trimester onwards and *IGF2DMR* methylation seemed to be slightly more profound in girls. This finding is in contrast to previous research which demonstrated that boys may be more susceptible to detrimental exposures than girls [23, 18]. The first days after conception are important for the reprogramming of the epigenome. A limitation of our study is that we were not able to study difference in preconception and postconception smoke exposure on the methylation of the *IGF2*/*H19* locus. This warrants further research.

We have focused on the hypotheses that epigenetic alterations mediate the association between maternal tobacco smoking and adverse birth outcomes. Our study supports the notion that DNA methylation can be one of the mechanisms through which tobacco smoking impairs fetal growth. As nicotine crosses the placenta, it can directly harm the fetus [24]. Cigarette smoke has been shown to contain high levels of reactive oxygen species (ROS) causing oxidative stress [25]. DNA ROS damage can interfere with the binding of DNA methyltransferases (DNMTs) to the DNA, resulting in DNA hypomethylation [26]. Tobacco smoking can also indirectly damage the fetus through the effects on the placenta. Nicotine causes vasoconstriction and thus can impair placental function [27]. Increased vasoconstriction will limit blood flow and thus the provision of nutrients to the fetus, which could be compared to the effects of exposure to famine previously linked to epigenetic effects [9].

Mothers exposed to passive cigarette smoking show increased risks of adverse birth outcomes [28, 29]. Therefore, the strength of our study is that we were able to investigate direct intrauterine effects of maternal smoking by taking into account the effect of prenatal smoking of both parents. The effect of paternal smoking was not statistically significant and smaller than the effect of maternal smoking. This suggests that the observed effect is largely due to direct intrauterine effects of maternal smoking and not to unmeasured environmental factors as the mother and father are likely to share their environment, which is substantiated by a recent study [30].

DNA methylation is believed to be relatively stable throughout life, but these patterns are reprogrammed significantly during fetal and early postnatal life [7]. Therefore, it is assumed that the epigenome is susceptible for derangements during developmental stages when adverse prenatal environmental exposures can permanently alter methylation in the offspring. The periconception period is of particular importance, as epigenetic reprogramming takes most place during this period [7]. Our findings support the notion that changes in DNA methylation at the *IGF2DMR* are being established during the periconception period, as the methylation levels of mothers who quit smoking during the first trimester showed a tendency to be lower than in non-smokers. The non-significance may also be explained by small numbers of mothers who quit smoking during the first trimester. However, a larger effect was observed in mothers who continued to smoke during pregnancy, indicating that these effects are not limited to the periconception period.

### Strengths and limitations

This study was embedded in a large cohort from which a selection of Dutch newborn were studied. Selective participation did occur to some degree, as participating mothers were younger, more often single and smokers, and newborns were more often boys. This selection could have affected the external validity and generalizability of the study, especially when the associations would differ between the study population without and with the excluded newborns. This is difficult to ascertain, as the associations in the excluded group are unknown.

The study was based on a prospective data collection starting in early pregnancy. Detailed information was available on prenatal parental tobacco smoking habits, which enabled studying both trimester specific and dose-response associations. Although the use of questionnaires seems to be a valid method misclassification could have occurred, mostly leading to underreporting of tobacco smoking and therefore an underestimation of our estimates [31]. Lastly, although analyses were adjusted for potential confounders, the possibility of residual confounding cannot completely be excluded.

This study showed modest changes in DNA methylation in mothers who continued to smoke during pregnancy. Replication of these results is warranted. We were not able to assess whether the DNA methylation variations also resulted in changes in expression and long-term functional effects, although this has recently been shown in studies of others [32–34]. DNA methylation was measured in umbilical cord white blood cells and not in other tissues. It could be argued that DNA methylation patterns differ across various tissues [35, 36]. However, DNA methylation of imprinted genes such as *IGF2DMR*/*H19* is believed to be largely comparable in different tissues. For example, DNA methylation patterns of *IGF2DMR* in blood and in buccal cells and colon tissue showed moderate correlations [35, 37, 36]. We emphasize that it remains important to establish correlations between DNA methylation in peripheral tissues, e.g., blood, and tissues that are directly involved in the disease. However, in human epidemiological studies, it is commonly accepted to use blood or buccal samples due to relatively poor accessibility of other tissues.

## Conclusions

Our study shows an inverse association of maternal prenatal tobacco smoking and its dose and duration with *IGF2DMR* methylation in newborns. *IGF2DMR* methylation may mediate the association between maternal tobacco smoking and the risk of a newborn born SGA, supporting the notion that epigenetic alterations could be one of the mechanisms through which smoking affects fetal growth. The understanding how epigenetic control depends on early exposure may shed light on the link between fetal development and health over the life course. Our research emphasizes the importance to quit smoking preferably in the preconception period, although our data also shows that the advantageous effects of quitting smoking may not be limited to this period.

## Methods

### Design and study population

This study was embedded in the Generation R Study Rotterdam, the Netherlands, a population-based prospective cohort study from early pregnancy onwards [38]. The study has been approved by the Medical Ethical Committee of the Erasmus Medical Center in Rotterdam (MEC 198.782/2001/31). Written informed consent was obtained from all participating mothers for both maternal and child data. Mothers were enrolled during pregnancy, between 2001 and 2006. In total, 8880 mothers were enrolled during pregnancy.

For the present study, analyses were restricted to Dutch newborns, which were based on the country of birth of the parents and grandparents (*n* = 4882) [38]. In addition, newborns were selected with DNA extracted from umbilical cord white blood cells available (*n* = 3127). DNA methylation measurements were limited to 540 newborns due to logistic reasons. The current study is part of a project, in which we investigate the hypothesis that both SGA and children with ADHD have a shared causality in DNA methylation of especially imprinted fetal growth genes. The sampling strategy was to oversample the children born SGA or diagnosed with ADHD to improve the power of the analyses. Therefore, all infants born with a gestational age and sex-adjusted birth weight below −2 SDS (*n* = 69) were selected for analysis. Also, 92 children were included with ADHD based on parent interview Diagnostic Inventory of Screening Children or Child Behavior Checklist teacher report at age 6. Two of the infants with ADHD were also born with a gestational age and sex-adjusted birth weight below −2 SDS. The remaining 381 control newborns were randomly selected. Controls were born with a gestational age and sex-adjusted birth weight above −2 SDS. Of the 540 newborns, data on parental smoking habits was available in 93.7 % (*n* = 506). The total population for analysis was 506 children (65 newborns born SGA, 88 cases of ADHD, and 441 control newborns). Characteristics of the included and excluded mothers and newborns were compared. Included versus excluded mothers were younger (30.3 years versus 31.1 years, *P* value < 0.001), smoked more often during pregnancy (32.9 % versus 26.0 % , *P* value < 0.001), and were more often single (10.9 % versus 8.4 %, *P* value = 0.02). Included versus excluded newborns were more often male (58.7 % versus 49.7 (*P* value < 0.001)). However, included and excluded participants were similar in terms of maternal BMI, maternal educational level, parity, folic acid supplement and alcohol use, and APGAR score after 1 and 5 min (all *P* > 0.05).

### Prenatal parental tobacco smoking habits

Information regarding maternal tobacco smoking was obtained by self-administered questionnaires sent in each trimester of pregnancy. Active maternal tobacco smoking at enrolment was assessed by asking the mother whether she smoked during pregnancy (no tobacco smoking, first trimester only tobacco smoking, continued tobacco smoking during pregnancy). This questionnaire was sent to all mothers, regardless of their gestational age at enrolment. In the second and third questionnaires, mothers were asked whether they smoked in the previous 2 months (no, yes). Mothers who reported that they did not smoke or only smoked during the first trimester, but still reported smoking in the second or third questionnaire, were classified as continuous smokers. Among the mothers who continuously smoked during pregnancy, the number of cigarettes were assessed and classified as (1) less than five cigarettes a day and (2) five or more cigarettes a day. Dose-response analyses for continuous smokers were based on the third trimester questionnaires. Prenatal paternal tobacco smoking was assessed at enrolment by asking the mother whether the father smoked during pregnancy (no, yes, do not know). Similar information was provided by the father in a subset of the participants (*n* = 433). Agreement between the two assessments was good (sensitivity = 92 %, specificity = 95 %). As more data was available from the mother questionnaire compared to the father questionnaire (6.7 % missing versus 19.8 % missing, respectively), data obtained from the mother’s questionnaire was used.

### Assessment of DNA methylation

Genomic DNA was isolated from cord blood samples at birth as previously described [39]. We assessed DNA methylation of two imprinted loci, namely *IGF2DMR* and *H19DMR*. These loci were chosen based on their previously shown features of epigenetic regulation and their susceptibility for to environmental exposures [40]. Details of the measured amplicons can be found in Additional file 4: Table S4. Isolated genomic DNA (500 ng) was treated with sodium bisulphite for 16 h using the EZ-96 DNA methylation kit (Shallow) (Zymo Research, Irvine, CA, USA), according to the manufactures’ protocol. Samples were randomly distributed on six 96-well plates. The bisulphite treatment was followed by polymerase chain reaction (PCR) amplification, fragmentation after reverse transcription, and analysis on a mass spectrometer, according to the manufactures’ protocol (MassARRAY EpiTYPER, Sequenom, Inc, San Diego, CA, USA). This generated mass signal patterns that were translated into quantitative DNA methylation levels of different CpG sites of the selected loci by MassARRAY EpiTYPER Analyzer software (v1.0, build1.0.6.88 Sequenom, Inc, San Diego, CA, USA) [41, 42]. Fragments containing one or more CpG sites were called CpG units. PCR and subsequent steps were performed in triplicate.

Data quality control for methylation consisted of exclusion of CpG units with too low or too high mass or CpG units with overlapping or duplicate RNA fragments (e.g., silent signals) were excluded from further analysis. Furthermore, at least two out of three of the replicate measurements per CpG unit had to be successful, the standard deviation of the duplicates or triplicates had to be ≤0.10, and the success rate per CpG unit had to be ≥75 %. Also, from previous research it is known that PCR bias can occur with quantitative DNA methylation measurements methods [43]. To address this potential problem, we created standard curves constructed from DNA with low and high methylation (EpigenDx, Worcester, MA, USA) in steps of 10 % methylation difference on both amplicons. CpGs showing irregularities were excluded from the analyses. Last, CpG units with interference of single nucleotide polymorphisms (CEU) with a frequency of >5 % were also excluded, as this could change the weight of the CpG unit and therefore interfere with the measurement. Details concerning the success rate of the amplicons can be found in Additional file 5: Table S5.

### Covariates

From self-administered questionnaires, data was available on maternal age and maternal educational level, parity, and folic acid supplement use before and during pregnancy. Maternal education level was assessed by the highest completed education and classified as (1) none/primary or “low”, (2) secondary or “medium”, and (3) college/university or “high”. Parity was classified as (1) nulliparous and (2) multiparous. Folic acid supplement use was categorized into (1) folic acid supplement use (pre- or postconception start) and (2) no folic acid supplement use. At enrolment (median 13.5 weeks, 90 % range 10.7–21.6), maternal weight and height were measured to calculate BMI (kg/m^2^). Information concerning date of birth, offspring sexm, and birth weight was obtained from community midwives and hospital registries.

### Statistical analyses

Differences in maternal or newborns characteristics between maternal tobacco smoking categories were tested using ANOVA and chi-square tests. Thereafter, linear mixed models were used to examine the associations between maternal or paternal tobacco smoking (independent variable) and DNA methylation (dependent variable). This model was chosen as it can account for correlation between CpG dinucleotides, incorporates relevant adjustments within the models, and has the ability to accommodate missing data. The restricted maximum likelihood method was used for the model fitting. DNA methylation was treated as a continuous variable. To achieve normality, DNA methylation was square root transformed. Outliers per CpG (>3SDS) were excluded from further analysis. For all analyses, subject/person identifier was added as random effect and bisulphite batch and CpG site were added as fixed effects. In the crude analyses, maternal and paternal tobacco smoking were both entered as a fixed effects in separate models. In the adjusted analyses, potential confounders were additionally entered to the model at the same time as fixed effects.

Since maternal and paternal smoking are correlated, investigating paternal smoking among all mothers would overestimate the effect of paternal smoking if direct intrauterine mechanisms were present. Therefore, the association between paternal smoking and DNA methylation was examined among mothers who did not smoke during pregnancy. Prenatal exposures have shown to alter *IGF2DMR* methylation differently according to sex. [18] Therefore, the association between maternal tobacco smoking and DNA methylation was assessed in both boys and girls. The analyses were also repeated with exclusion of the ADHD (*n* = 88) cases. Two newborns were classified as both ADHD and SGA.

Additionally, we performed a mediation analyses to investigate whether DNA methylation of either *IGF2DMR* or *H19* mediates the association between maternal tobacco smoking and the risk of a newborns being born SGA. The direct and indirect effects between the dependent (SGA) and independent variable (maternal tobacco smoking) as well as the mediator (DNA methylation) were tested (Fig. 1) using a bootstrap approach (1000 samples) described by Preacher and Hayes [44].

Missing data of potential confounders (maternal educational level (0.6 %), maternal BMI (0.2 %), and maternal folic acid supplement use (13.2 %) were completed using the Markov-Chain-Monte-Carlo multiple imputation technique [45]. Ten imputed datasets were created. The linear mixed model analyses were performed using the data measured in triplicates including imputed missing data. Overall, the effect estimates using the imputed dataset were slightly smaller compared to the original dataset. All analyses were performed using the Statistical Package for the Social Sciences version 21.0 for Windows (SPSS Inc, Chicago, IL, USA).

## Additional files

Additional file 1: Figure S1. Graphical representation of the *IGF2DMR* locus. Additional file 2: Figure S2. Graphical representation of the Methylation levels of *IGF2DMR* and *H19*. Additional file 3: Table S3. Parental tobacco smoking habits and DNA methylation, with exclusion of ADHD cases. Results from linear mixed model analyses with maternal or paternal tobacco smoking as independent variable and DNA methylation as dependent variable. ^1^Analyses were performed with square root transformed methylation data and values are presented as regression coefficients (95 % confidence interval). Analyses on paternal smoking were restricted to non-smoking mothers. Additional file 4: Table S4. Details of measured amplicons and PCR primers. ^1^ Genome built: GRch 37.67. ^2^ Forward and reverse primer that will amplify the bisulphite converted genomic DNA. According to the MassARRAY EpiTYPER technology, taqs were added to the 5'end of the primers. Forward primer: 10mer spacer tag is added at the 5′ primer end with the following sequence: 5′-AGGAAGAGAG + primer. Reverse primer: T7 promoter is added to the 5′ primer end with the following sequence: 5′-CAGTAATACGACTCACTATAGGGAGAAGGCT + primer. Additional file 5: Table S5. Details quality control.

## Abbreviations

ADHD: attention deficit hyperactivity disorder
BMI: body mass index
DMR: differentially methylated region
DNA: deoxyribonucleic acid
DNMT: DNA methyltransferases
IGF2: insulin growth factor-2
PCR: polymerase chain reaction
ROS: reactive oxygen species
SDS: standard deviation score
SGA: small for gestational age
