# Supplementary figures and images for: Prenatal parental tobacco smoking, gene specific DNA methylation, and newborns size: the Generation R study
Source: Clin Epigenetics. 2015 Aug 11;7(1):83. doi: 10.1186/s13148-015-0115-z (PMC4531498; doi:10.1186/s13148-015-0115-z)

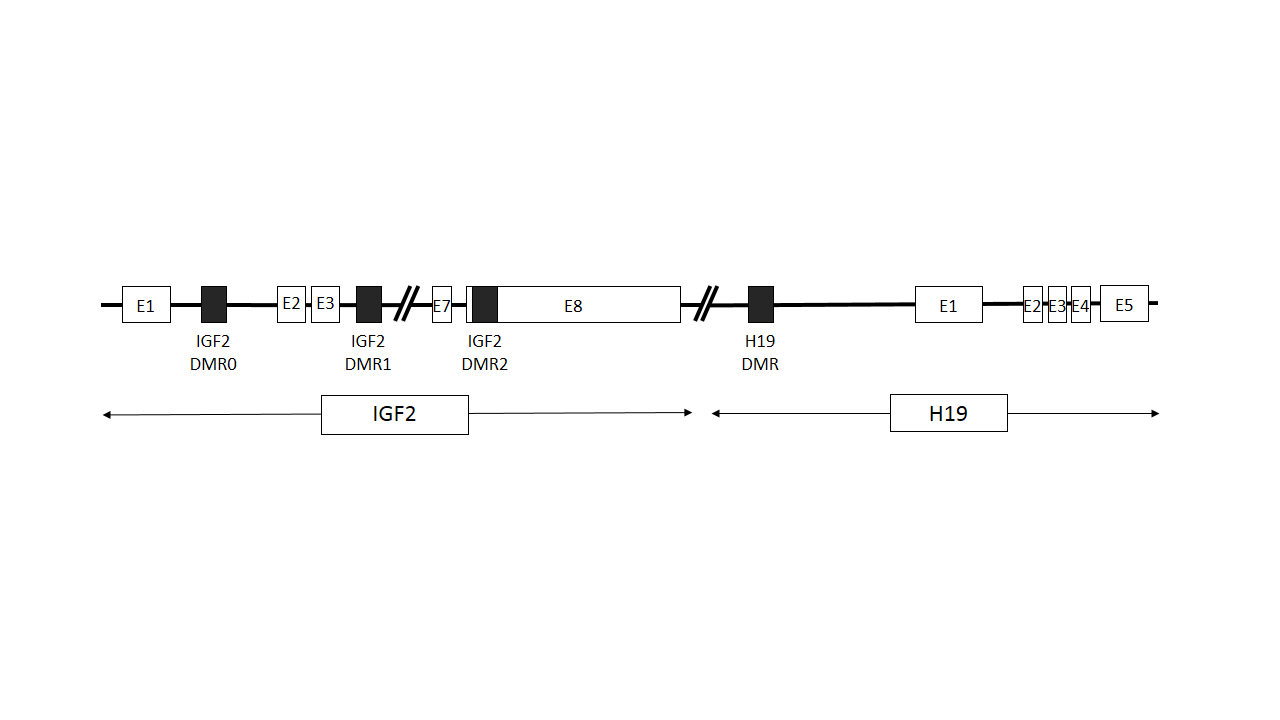

Supplement: Additional file 1: Figure S1. — Graphical representation of the IGF2DMR locus. [file 13148_2015_115_MOESM1_ESM.tiff]

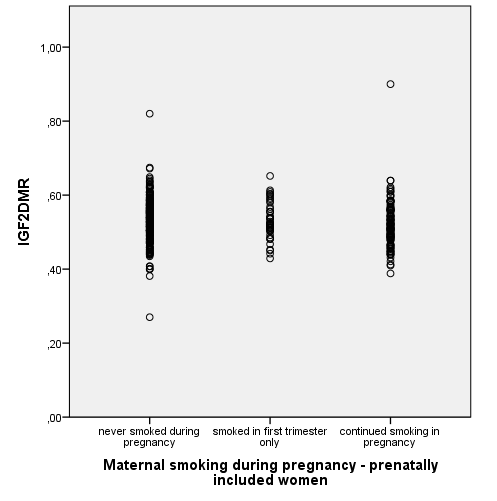


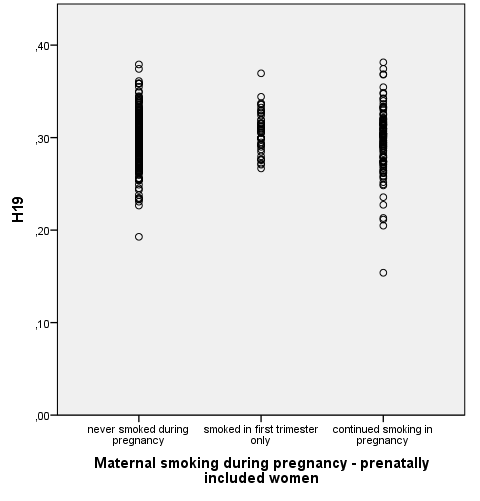

Supplement: Additional file 2: Figure S2. — Graphical representation of the Methylation levels of IGF2DMR and H19. [file 13148_2015_115_MOESM2_ESM.docx]
