# Supplementary material for: Prenatal parental tobacco smoking, gene specific DNA methylation, and newborns size: the Generation R study
Source: Clin Epigenetics. 2015 Aug 11;7(1):83. doi: 10.1186/s13148-015-0115-z (PMC4531498; doi:10.1186/s13148-015-0115-z)
Supplement: Additional file 3: Table S3. — Parental tobacco smoking habits and DNA methylation, with exclusion of ADHD cases. Results from linear mixed model analyses with maternal or paternal tobacco smoking as independent variable and DNA methylation as dependent variable. 1Analyses were performed with square root transformed methylation data and values are presented as regression coefficients (95 % confidence interval). Analyses on paternal smoking were restricted to non-smoking mothers. [file 13148_2015_115_MOESM3_ESM.pdf]

	IGF2DMR methylation			*H19* methylation		
	bèta[1]	95% CI	P-value	bèta[1]	95% CI	P-value
**<u>MODEL 1:</u> adjusted for correlations between CpG sites, bisulphite batch, gestational age at birth**						
Maternal tobacco smoking						
No (n=277)		reference			reference	
First trimester only (n=37)	-0.82	-1.94; 0.31	0.154	0.48	-0.37; 1.33	0.265
Continued smoking, all (n=104)	**-1.13**	**-1.87; -0.39**	**0.003**	-0.43	-0.99; 0.13	0.135
<5 cigarettes per day (n=34)	**-1.25**	**-2.43; -0.08**	**0.037**	**-0.92**	**-1.80; -0.04**	**0.041**
≥5 cigarettes per day (n=51)	-0.93	-1.94; 0.07	0.068	-0.18	-0.91; 0.56	0.637
P for trend		**0.021**			0.300	
Paternal tobacco smoking						
No (n=176)		reference			reference	
Yes (n=100)	-0.19	-0.98; 0.60	0.639	0.17	-0.39; 0.73	0.550
<5 cigarettes per day (n=48)	-0.26	-1.30; 0.78	0.621	0.16	-0.58; 0.89	0.679
≥5 cigarettes per day (n=51)	-0.05	-1.06; 0.96	0.922	0.16	-0.56; 0.89	0.658
P for trend		0.819			0.603	
**<u>MODEL 2:</u> model 1 + maternal characteristics (age, educational level, parity, BMI, periconceptional folic acid supplement use) and fetal gender**						
Maternal tobacco smoking						
No (n=277)		reference			reference	
First trimester only (n=37)	-0.89	-2.04; 0.25	0.127	0.40	-0.46; 1.25	0.366
Continued smoking, all (n=104)	**-1.18**	**-1.98; -0.37**	**0.004**	**-0.62**	**-1.24; -0.00**	**0.049**
<5 cigarettes per day (n=34)	**-1.30**	**-2.50; -0.10**	**0.033**	**-0.93**	**-1.82; -0.03**	**0.043**
≥5 cigarettes per day (n=51)	-0.81	-1.88; 0.26	0.136	-0.32	-1.11; 0.46	0.420
P for trend		**0.044**			0.184	
Paternal tobacco smoking						
No (n=176)		reference			reference	
Yes (n=100)	-0.19	-1.01; 0.63	0.646	0.05	-0.51; 0.62	0.860
<5 cigarettes per day (n=48)	-0.23	-1.29; 0.83	0.670	0.14	-0.59; 0.86	0.712
≥5 cigarettes per day (n=51)	-0.07	-1.14; 1.00	0.895	-0.07	-0.82; 0.67	0.849
P for trend		0.803			0.958	

Table S3: Parental tobacco smoking habits and DNA methylation, with exclusion of ADHD cases

Results from linear mixed model analyses with maternal or paternal tobacco smoking as independent variable and DNA methylation as dependent variable. [1]Analyses were performed with square-root transformed methylation data and values are presented as regression coefficients (95% confidence interval). Analyses on paternal smoking were restricted to non-smoking mothers.
