# Supplementary material for: Prenatal parental tobacco smoking, gene specific DNA methylation, and newborns size: the Generation R study
Source: Clin Epigenetics. 2015 Aug 11;7(1):83. doi: 10.1186/s13148-015-0115-z (PMC4531498; doi:10.1186/s13148-015-0115-z)
Supplement: Additional file 4: Table S4. — Details of measured amplicons and PCR primers. 1 Genome built: GRch 37.67. 2 Forward and reverse primer that will amplify the bisulphite converted genomic DNA. According to the MassARRAY EpiTYPER technology, taqs were added to the 5'end of the primers. Forward primer: 10mer spacer tag is added at the 5′ primer end with the following sequence: 5′-AGGAAGAGAG + primer. Reverse primer: T7 promoter is added to the 5′ primer end with the following sequence: 5′-CAGTAATACGACTCACTATAGGGAGAAGGCT + primer. [file 13148_2015_115_MOESM4_ESM.pdf]

Gene	Genomic location[1]	Number of CpG units assessed	Primer sequence[2]	Source
Insulin-like growth factor 2 (IGF2)	Chr 11: 2169458-2169796	3 CpG units (4 CpG sites)	F: TGGATAGGAGATTGAGGAGAAA<br>R: AAACCCCAACAAAAACCACT	Heijmans, 2007
H19	Chr 11: 2019371-2019784	10 CpG units (13 CpG sites)	F: GGGTTTGGGAGAGTTTGTGAGGT<br>R: ATACCTACTACTCCCTACCTACCAAC	Heijmans, 2007

Table S4: Details of measured amplicons and PCR primers

[1] Genome built: GRch 37.67

[2] Forward and reverse primer that will amplify the bisulphite converted genomic DNA. According to the MassARRAY EpiTYPER technology, taqs were added to the 5'end of the primers. Forward primer: 10mer spacer tag is added at the 5’ primer end with the following sequence: 5’-AGGAAGAGAG + primer. Reverse primer: T7 promoter is added to the 5’ primer end with the following sequence: 5’-CAGTAATACGACTCACTATAGGGAGAAGGCT + primer
