# Supplementary material for: Prenatal parental tobacco smoking, gene specific DNA methylation, and newborns size: the Generation R study
Source: Clin Epigenetics. 2015 Aug 11;7(1):83. doi: 10.1186/s13148-015-0115-z (PMC4531498; doi:10.1186/s13148-015-0115-z)
Supplement: Additional file 5: Table S5. — Details quality control. [file 13148_2015_115_MOESM5_ESM.pdf]

Locus/unit	Reason for exclusion	Success rate (if included)
IGF2 DMR		
IGF2_01	Excluded due to rs3741208	
IGF2_02	Excluded due to rs3741209	
IGF2_03		91.8%
IGF2_04		94.1%
IGF2_05	Excluded due to rs4930041	
IGF2_06.07		94.9%
IGF2_08	Excluded due to silent signal	
H19		
H19_01	Excluded due to duplicate H19_16 and silent signals	
H19_02		95.6%
H19_03-05	Excluded due to rs117916983, overlap H19_11 and silent signals	
H19_06	Excluded due to silent signals	
H19_07	Excluded due to silent signal	
H19_08	Excluded due to silent signal	
H19_09.10		95.0%
H19_11	Excluded due to overlap H19_03-05 and silent signals	
H19_12		95.4%
H19_13		95.1%
H19_14.15		96.7%
H19_16	Excluded due to duplicate H19_1 and silent signals	
H19_17		95.3%
H19_18.19		95.6%
H19_20		95.6%
H19_21	Excluded due to low mass and silent signal	
H19_22	Excluded due to silent signal	
H19_23	Excluded due to low mass	
H19_24	Excluded due to >25% missing	
H19_25		94.7%

Table S5: details quality control
